# Application of a method for the sustainable planning and management of ground source heat pump systems in an urban environment, considering the effects of reciprocal thermal interference

**DOI:** 10.12688/openreseurope.14665.2

**Published:** 2022-11-25

**Authors:** Marco Belliardi, Linda Soma, Rodolfo Perego, Sebastian Pera, Eloisa Di Sipio, Angelo Zarrella, Laura Carnieletto, Antonio Galgaro, Borja Badenes, Riccardo Pasquali, David Bertermann, Burkhard Sanner

**Affiliations:** 1 University of Applied Sciences of Southern Switzerland, Mendrisio, 6850, Switzerland; 2 University of Padua, Padova, 35131, Italy; 3 Institute of Information and Communication Technologies, Universitat Politècnica de València, Valencia, Valencian Community, 46022, Spain; 4 Terra GeoServ Ltd, Wicklow, A98 N6X4, Ireland; 5 University of Erlangen-Nuremberg, Erlangen, 91054, Germany; 6 UBeG GbR, Wetzlar, 35580, Germany

**Keywords:** geothermal energy, GSHP, BHE, thermal interference, planning

## Abstract

The “Most Easy, Efficient and Low Cost Geothermal Systems for Retrofitting Civil and Historical Buildings” (GEO4CIVHIC) project aims to accelerate the deployment of shallow geothermal systems for heating and cooling purposes when retrofitting existing and historical buildings. Analyzing the implementation process of borehole heat exchangers (BHEs), allows the understanding of how to promote the long-term sustainability of shallow geothermal energy systems. The thermal interference between BHE systems represents a problem, especially due to the increasing deployment of this technology and its spread in densely built-up areas.

The main goal of this paper is to propose a conceptual model and to apply this to different case studies. The methodology includes phases to adopt an integrated approach for preventing long term thermal interference in neighbouring borehole heat exchangers, by providing management strategies and technical suggestions for design and operation.

The method developed follows the following steps: 1) literature review to determine what are the main drivers for thermal interference between shallow geothermal systems, in the context of the GEO4CIVHIC project case study sites; 2) to create a conceptual model to limit thermal interference at both design and operational phases; 3) to apply the developed method to real and virtual case studies in countries with different regulatory frameworks and to test its main strengths and weaknesses. The application of this conceptual model to specific case studies provides evidence of critical planning and operational characteristics of GSHP systems and allows the identification of measures to mitigate impacts of thermal interference to be identified.

## Introduction

In the last decades, the European geothermal heat pump market has continued to increase, reaching 2.1 million operating units during 2020 ^1^. Despite the COVID-19 pandemic decreasing the sales of ground source heat pumps (GSHPs) in some countries, around 100,000 were sold in countries with Nordic or Alpine climates such as Sweden, Germany, and Netherlands. These countries cover half of European sales ^1^ where high GSHP penetration rates prevailed. Moreover, Switzerland represents one of the first five countries using shallow geothermal energy where borehole heat exchanger (BHE) systems are the dominant application ^2^ in terms of installed capacity of thermal power per population (MW _th_/population), land area (MW _th_/km ^2^) and annual energy use for area (TJ/yr/km ^2^) ^2^.

Despite the increase in global market sales of heat pumps in the latest years, GSHPs maintain an average of 90 thousand units sold in the period 2014–2018 ^3^. The use of vertical ground source heat exchangers (GSHEs) has increased in urban and built environments. For example, in Stockholm, more than one third of all single-family houses, not connected to district heating, have a ground source heat pump ^4^.

The EU’s energy policy is focused on improving efficiency ^5^ and the use of the renewable energy ^6^. These two factors have to be addressed in the context of the sustainability, which requires that the resource must be available even in the long term ^7^.

In urbanised areas, a combination of district heating and stand-alone GSHP systems can result in minimum primary energy demand to supply heat to all the users. Nevertheless, different constraints on GSHP such as thermal interferences between neighbouring systems, must be considered ^8, 9^.

The objective of this article is to present recommendations, starting from the state of the art used to find a method to address thermal interference and applying this method to the case studies collected and analysed during the GEO4CIVHIC project ^10^ by giving indications to resolve or prevent possible thermal interference between nearby geothermal systems. These preventative recommendations would help to maximise and guarantee long term efficiency for all geothermal systems. Since the end of the 20 ^th^ century, studies have confirmed that if geothermal systems are near to each other, there are thermal interferences that can have important effects on the sustainable operation over time, leading to inefficiencies and potential system failure ^8, 11^. For these reasons, interference must be considered during the planning phase of any geothermal project. The incorrect management of the resource can generate problems: the thermal and physical interference, malfunction and blocking operation of the systems and ground freezing ^12, 13^.

## Methods

**Figure 1.**
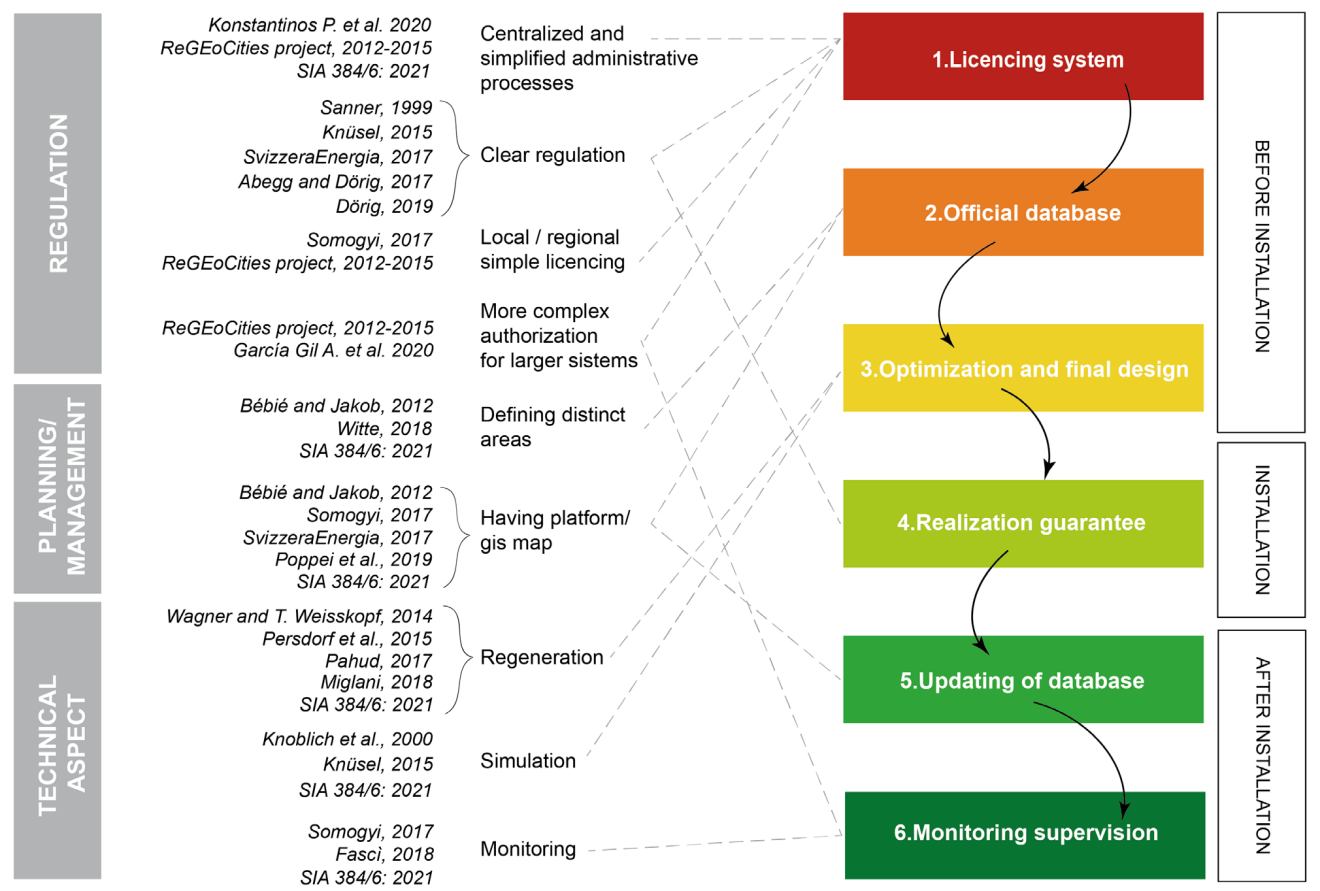
Schematic of the research and main technical references considered related to thermal interference.

The most important key factors necessary for the correct planning of GSHP installations are presented on the right side of the diagram in Figure 1. These factors were chosen based on the analysis of the data and of the different regulatory procedures implemented in several countries that were individually assessed as part of the GEO4CIVHIC project and described in a public deliverable ^14^.

Based on previous literature review described in 14, the present research updates and present a literature review of the state of the art related to interferences between geothermal systems with BHE ( Figure 1).

The literature review involved in particular:

- national and international technical reports (considering documents in English, German, French and Italian);
- the Scopus database using specific keywords in the field of proximity of shallow geothermal systems (e.g.: proximity of borehole heat exchangers system, ground source heat pumps in neighbourhood, geothermal interference, geothermal installations in dense urban areas);
- documentation the authors of this article have accessed through participation and collaboration with professional associations, working groups, and research programs in the shallow geothermal context.

The approach was not only to include the technical standpoint, that is usually considered in papers and technical studies, but also considering other key factors that are also necessary in the correct installation of a geothermal plant (i.e., administrative procedures, system location tracking and monitoring, etc.).

**Figure 2.**
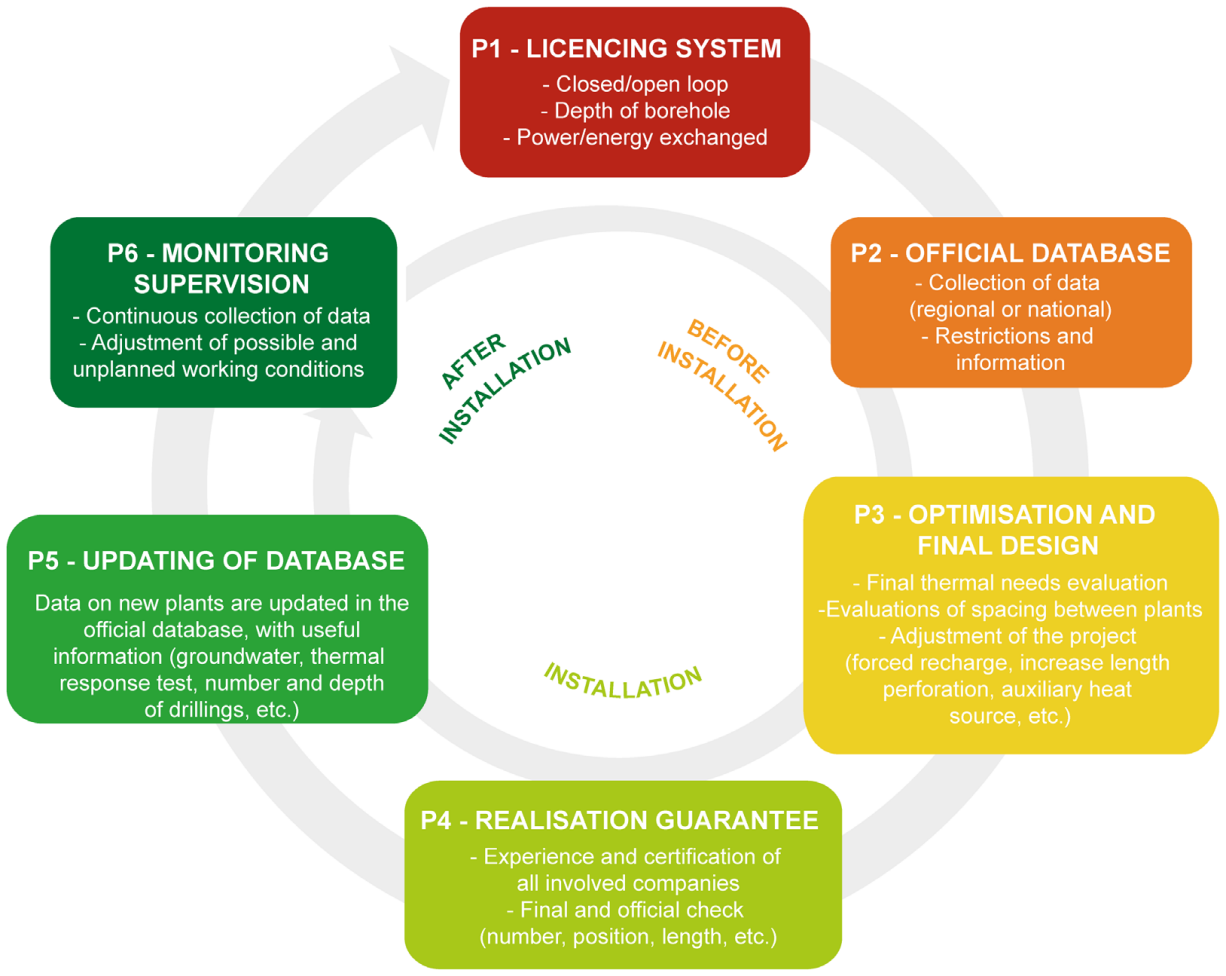
Conceptual model (CM) considering the complete and ideal phases for the realisation of a ground source heat pump system.

A set of basic chronological steps were structured to define all the stakeholders involved in the licencing, planning, design, construction, and operation of geothermal systems. A conceptual model, that simplifies the major phases of the BHE systems installation was elaborated and is presented as a 6-phase circular procedure (P1, P2, etc.) in Figure 2. The licencing system (P1), the presence of an official database where data are collected (P2) and the optimisation phase (P3) are the first three steps of the procedure. An official database is a geo-referenced list of existing installations, managed by a central authority, with related and dedicated information, such as the position, number and depth of boreholes drilled, the thermal power and energy exchanged, and the year of installations. After the realization of the geothermal system, the correct implementation of the project must be verified (P4). After this verification of the implementation, the updating of the database (P5) is essential to ensure that data recorded corresponds to the information declared as part of the planning, installation and optimisation phases. This is important for the future planning of nearby installations. The last phase of the procedure (P6) includes monitoring and supervision. This last step allows the verification of the efficiency of the operating system, the updating of the licensing system and the database and finally to evaluate the need for any possible adjustments.

The conceptual model was then applied to the GEO4CIVHIC virtual and real case study sites with a semi-qualitative approach ^14^. The possibility to apply the model on case studies located in different European countries and frameworks allowed bringing to light more critical points to consider in the European policy to prevent future thermal interference effects.

## A conceptual model for geothermal planning

As mentioned in 8, since 1999 “how sustainable is shallow geothermal energy?” has been a question needing to be answered. Thanks to the monitored data, the importance of the correct sizing was stressed as a fundamental aspect ^8^. At that time, the importance of the cooperation between different stakeholders and professionals involved operating in the geothermal sector already appeared clear, as well as the presence of a political guideline at European level. Specific software for simulation has been used to counteract the mutual influence in heat extraction, providing longer probes lengths in the case of dense buildings (i.e.: EED ^15^, TRNSBM ^16^) ^11^.

Knowledge of the geothermal potential of an area is very useful to sustainably exploit shallow geothermal energy ^17^ and to perform technical and economical pre-feasibility assessments for new systems ^18^. Planning with an integrated strategy is also necessary to manage conflicts between resources in the urban planning of the underground ^19^.

According to the multidisciplinary nature of the activities involved (geothermal energy resources, heating/cooling facilities and environmental concerns) ^20^, clear regulations are necessary to implement and realise the BHE systems ^21– 23^. The licencing system should be as simple ^24^ and centralized as possible in such a way as to facilitate end-users interested in installing BHE systems. For large systems, a more complex authorisation is more appropriate, including risk assessment, environmental impact assessment, authorisation and subsequent monitoring ^25^. The presence of a public platform or geographic information system (GIS) based map can be very useful ^12, 22, 24, 26, 27^. In Europe there is a lack of a common regulative framework, preventing an easy and efficient application of BHE ^28^. Some countries (e.g., Netherland and Switzerland) propose the definition of different areas based on the energy demands of the buildings to manage future installations ^29^. Measures need to be taken to prevent thermal interference in densely populated areas that take into account the presence of neighbouring boreholes during the design phase ^9^. In addition, some GIS based methods to estimate the technical potential of BHE systems considering potential thermal interference have been developed ^30, 31^.

In some cases, simulation of BHE systems ^11, 32^ and regeneration could be a way to prevent interference ^33– 36^. Monitoring should be implemented ^24, 37^ in order to observe if the system is operating as planned and, if not, allow any correction or changes to be made. The updated Swiss standard ^12^ also underlines the importance of measurements, which should observe the thermal behaviour of the BHE over time, allowing the GHSP system to be optimised if needed.

The diagram in Figure 1, brings together information from different publications and groups them into macro-areas, emphasising that very different aspects (regulation, planning/management, technical aspect) have a direct impact on the possible thermal interaction between geothermal systems. The diagrammatic representation of the review analysis aims to systematically describe the topics that should be introduced, at a regional or national level, for an efficient management of GSHP systems.

Based on the review analysis completed as part of the previous report underlying this work ^14^, it was possible to highlight key points that have been simplified and integrated to propose a circular licensing system as a 6-phase conceptual model ( Figure 2). The circular nature shows the connection between the steps and demonstrates that, if any conditions are not compatible with the previous steps, the process must be repeated. For example, if the monitoring (P6, Figure 2) shows parameters out of the range addressed in phases 1 to 5 (e.g., temperatures or heating requirements) it is necessary to check that the licensing system requirements for the operating system are respected.

The **P1-Licencing system** phase is necessary to check if the project fits the existing requirements, which should be clear, rigorous, and inclusive of all relevant aspects for the BHE system installation process. The licencing system should be simple and not too onerous or, at least, not for smaller systems that normally have a small thermal impact. All relevant aspects in the field of interference should also consider. To avoid misunderstandings between private individuals, professionals and public administrations, the licensing system should be coherent and approved at least at national level, whilst its management can be delegated at regional level.

The data collected in the first phase (licensing system), should be uploaded as part of the second phase ( **P2-Official database**) to a common geo-localized database to facilitate improved territorial management of the BHE systems. This database should be implemented, managed, and maintained by national or regional public offices, depending on the specific regulation. The geo-localized database allows the valorisation of information, an increase in knowledge and reduction in data gaps, a better monitoring of deployment trends of BHE systems, preventing interference and offering clear guidance for GSHP systems in areas where legislative restrictions occur. This database makes the development of systems more efficient and rapid, increasing awareness and management of past and future installations., GIS is fundamental for the sustainability of every BHE project, as demonstrated by the Swiss standard ^12^ where the necessary data including: drilling location, depth, expected annual amount of energy extracted and annual amount of heat injected are recorded.

The third step is the **P3-Optimisation and the final design phase** an optimal system allows the temperature of the heat transfer fluid and the minimum number of BHE installed to be satisfied simultaneously. The optimisation of the system must, therefore, make it as efficient as possible, from an economic, energetic, and from an environmental standpoint. The thermal requirements must be accurately estimated and it must remain the same over the years of operation, or in any case, their modification should not penalize the overall functioning of the system, in order to ensure long-term, sustainable system operation. Moreover, the number and layout of drilled boreholes is a fundamental parameter to correctly evaluate the geothermal system operation. The proximity of boreholes close to the property boundaries can increase the probability of thermal interference with neighbouring systems in dense urban areas ^12^. Finally, where other geothermal plants are in close proximity, it is necessary to evaluate solutions that can cancel or limit the negative effects of thermal interference between these plants.

The fourth step is the **P4-Realization guarantee**: once the technical aspects are defined, one of the steps that is often missing is a verification that the project is carried out and installed as described, and therefore all the specifications considered during phase 3 are put into practice. The realization guarantee can be verified in two ways. The first is that the companies responsible for the construction and installation of the system, are certified or that they are able to carry out the work using state of the art methods. This aspect can be improved through training of professionals with regular courses and updates in techniques and construction materials. The second is that competent authorities verify, during the construction phase, that the implementation is realised in accordance with P1.

The fifth step ( **P5-Updating of database**) requires that variations in the original project planned due to unforeseen ground conditions during the drilling and construction phase are recorded. Impediments related to some geological or aquifer water pressures issues may force the length, number or location of the perforations to be reconsidered. For these reasons, updating the information on the databases, after the implementation phase is essential to ensure that the correct data is present. When the data is not updated, the information in the database remains incorrect and consequently erroneously influences future considerations in the planning and recommendation for deploying GSHP systems.

The sixth phase ( **P6-Monitoring phase**) allows any discrepancies from what was initially planned to be identified. If, during the operational phase, the planned conditions are not respected, it is possible to subsequently adapt the system and to repeat the licensing procedure (P1) evaluating the compliance with the regulations and the subsequent steps. For large BHE systems, monitoring equipment that tracks at least the flow and return temperatures from the probes and the thermal regeneration of the ground has to be recorded ^12^.

## Application of the model to the real and virtual cases

**Figure 3.**
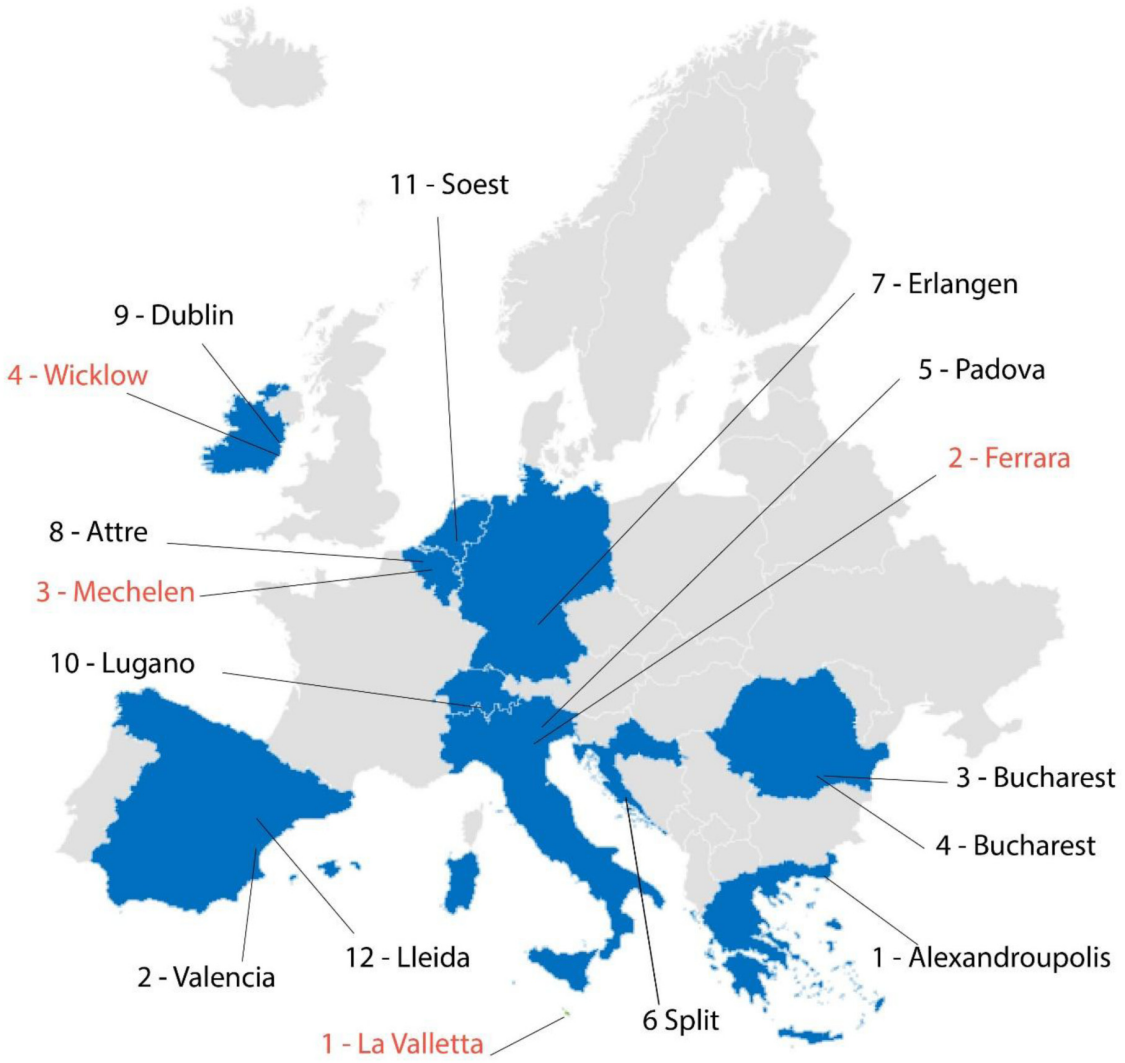
Maps of the real (red labels) and virtual (black labels) case studies of the GEO4CIVHIC project.

Within the framework of the GEO4CIVHIC project, 16 case studies, located in several European countries, were examined ( Figure 3). These consist of 4 real and 12 virtual cases studies. The virtual cases are existing buildings where the technology developed in GEO4CIVHIC project has been applied in a virtual way by means of simulations. On the other hand, in the real cases the technology has been trialled and installed through the completion of new systems. The assessment of the licensing and regulatory systems with respect to thermal interference has not differentiated between real and virtual case, where the study has equally considered local regulations and conditions.

Each one of the 4 real and 12 virtual case studies were analysed and assessed using the proposed 6-phase conceptual model ( Figure 2). For the details of the single case studies, see chapter 2 of the deliverable D6.3 of the GEO4CIVHIC project ^14^, where each case study is described.

**Figure 4.**
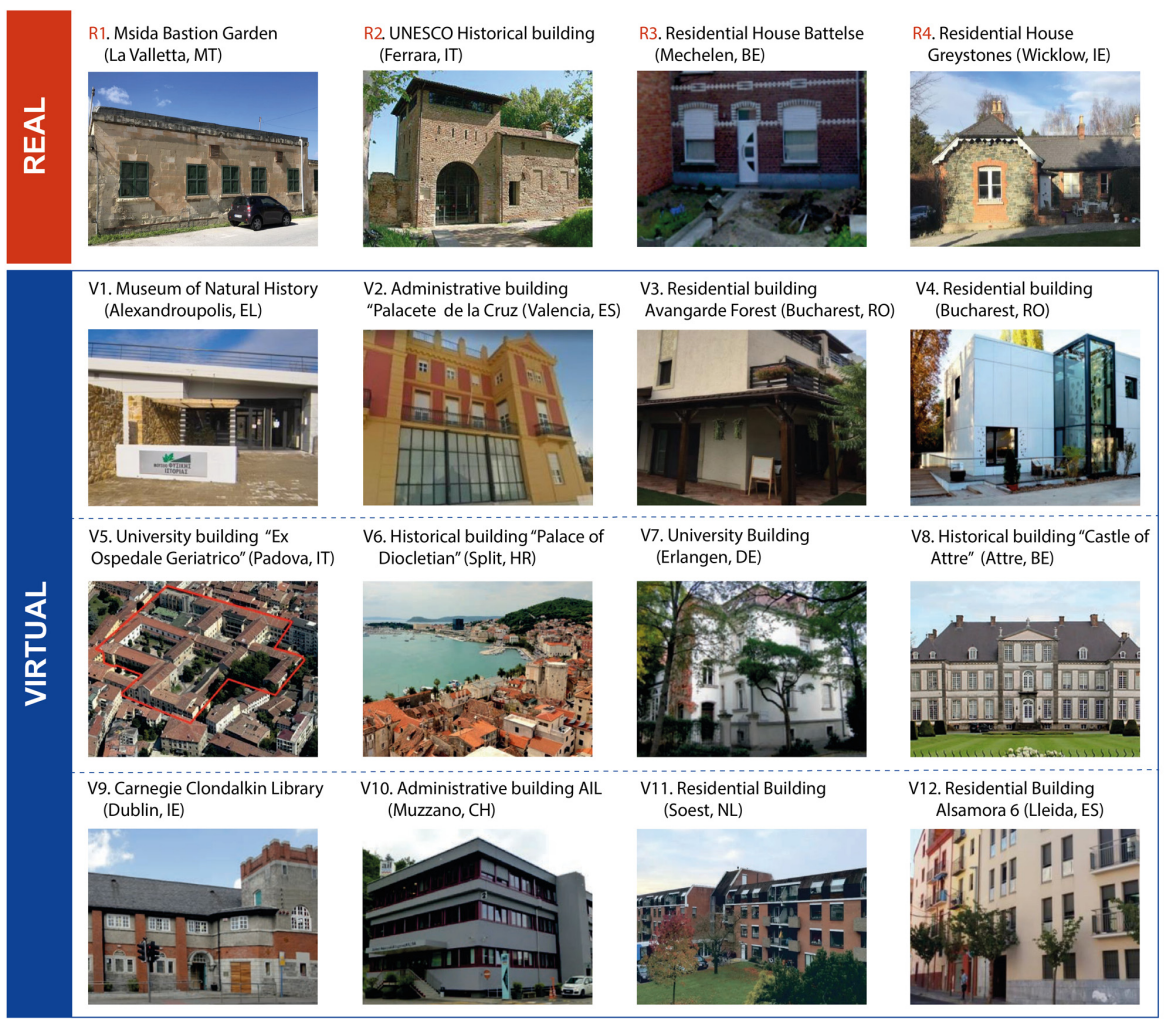
Real and virtual case studies of the GEO4CIVHIC project.

Figure 4 shows real and virtual case studies, for details of the single case studies, see deliverable 6.3 ^14^.

Each phase of the conceptual model was applied to each case study, by assigning a qualitative score from 0 to 3 (with 0 = not implemented at all, 3 = fully implemented) considering the following requirements:

- Score = 0 : the phase is not implemented at all;
- Score = 1 : the phase is only partially implemented, but can be significantly improved;
- Score = 2 : the phase is implemented, but can still be partially improved;
- Score = 3 : the step is fully implemented in an optimal manner.

The following table shows the scores assigned to the case studies for each of the six phases, based on the data collection performed as part of the GEO4CIVHIC project.

**Table 1.** Scores assigned to the case studies for each of the six phases. Virtual case studies n°6 and n°12 (V6 and V12) provided data only for phase 1 (P1), while virtual case study n°5 (V5) provided data only for phase 1 and phase 6 (P1 and P6). References numbering R1-R4 and V1-V12 correspond to Figure 4.

	R1	R2	R3	R4	V1	V2	V3	V4	V5	V6	V7	V8	V9	V10	V11	V12
**P1**	3	2	3	0	3	0	1	2	2	0	3	3	0	3	3	0
**P2**	1	0	3	0	2	0	0	0			3	3	0	3	3	
**P3**	3	3	1	3	3	1	3	3			2	1	3	2	3	
**P4**	2	2	1	0	3	0	1	1			3	2	0	1	3	
**P5**	1	0	2	0	2	0	0	0			3	1	0	1	3	
**P6**	0	3	0	0	2	2	0	0	3		3	0	0	1	3	

A calculation has been performed for each phase, in order to indicate the level of detail to which a phase is performed in all the case studies analysed.

A percentage (P) has been calculated for each phase, using the following formula:


$$P_{j}=\frac{\sum_{i=1}^{n}S_{ij}}{n\cdot max(S)}\cdot \%$$

Where:

- j : specific phase analysed (from P1 to P6);
- i : specific case study analysed (from 1 to 16);
- s : scored assigned to a specific phase (j) and to a specific case study (i). This can vary from 0 to 3 (only integer numbers assigned);
- n : total number of case studies (16);
- max (s) : best score that a phase can reach (always 3).

**Figure 5.**
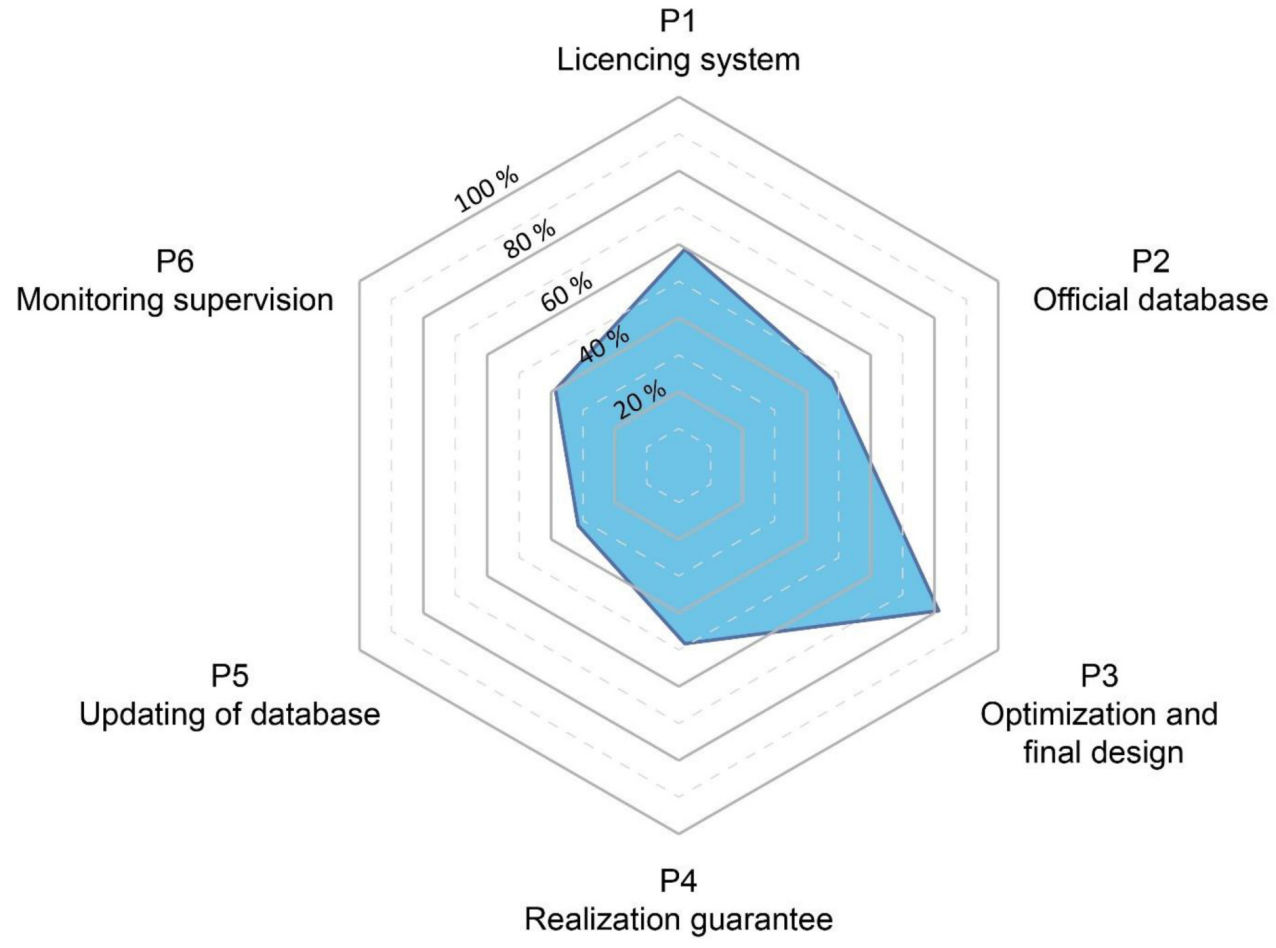
Results of the application of the model to the GEO4CIVHIC case studies.

The results obtained from the application of the model are shown in Figure 5, which describes an overall framework of the situation at each case study site. More details on the data used and underlying this figure are available in 14.

Many interesting aspects emerged by analysing the different real and virtual buildings located in several countries. However, it is not possible to generalize at national level, as the license systems can be at national, regional, or municipal level with different conditions and requirements, even in the same country.

The results based on the 16 cases show that typically the best addressed phase is the "optimisation and final design” phase (P3, Figure 5).

The licencing or permit system (P1, Figure 5) represents the second-best addressed phase (58%). However, even if many countries show that usually a general licencing system is present, the assessment has noted that sometimes these generic systems lack specific or clear licencing procedures especially for closed-loop systems. In addition, the drilling permission is not always required. This leads to a lack of knowledge of existing and future installation and prevents the implementation of adequate management and planning strategies that can promote the suitable development of resources.

When considering the possible interference prevention between neighbourhood systems, the results of the analysis from the 16 case studies shows that a minimum distance between plants is not always defined. The reason in some cases is that there was no clear legislative requirement for defining such distance. In some other cases, the use of adequate design tools and methodologies to optimise the planning and specification of the systems are not used (a detailed description regarding these aspects is available in 14 for each case study analysed). The lack of specific rules or tools to deal with interference, leads to unwritten rules which vary from case to case and inconsistencies with results that are not always easily assessable or demonstrable. Furthermore, without clearly defined and specific rules and regulations on interference, implies that specific analyses of possible interference effects are not required and therefore are not carried out.

The realization guarantee (P4, Figure 5) resulted in a score of 49% and the presence of a database (P2, Figure 5) a score of 46%. The realization guarantee (P4, Figure 5) along with the database (P2, Figure 5) are often missing and where these exist, the information is not publicly available. The database management is implemented at different levels (national, regional, municipal) when these are present. The lack of a database does not allow knowledge of the location and lengths of single drillings associated with GHSP systems. This hinders the realization of future new plants and the ability to manage new and previous installations. Moreover, obtaining information on previous installations is difficult to impossible, hence once the localisation of a single BHE is lost, a significant data gap in the future management and planning of resources is generated.

Finally, the monitoring phase (P6, Figure 5) results are 40% and the update of the database (P5, Figure 5) 33%. The monitoring phase (P6) is rarely present. Generally, the monitoring is considered expensive, and if the licencing system does not require this to be performed, it is not completed (especially for a closed loop systems). In other cases, monitoring is simply performed for control and to ensure that the system is performing adequately, but no historical data is collected or stored to facilitate further in-depth analysis.

The database update (P5, Figure 5) is the phase with the worst score, in some cases as a result of a lack of database implementation (P2, Figure 5), in others, because the final data are not reported or updated. The database update is important because data inserted at P2, during the planning, can differ from the ‘as built’ data after the final implementation and should be updated. For example, changes on the number of boreholes drilled, on their length and position can significantly influence the overall thermal physics in the subsoil.

## Conclusions

The deployment of geothermal heat pumps within Europe is extremely heterogeneous from one country to another; sometimes with a high degree of variability between regions of the same country. In this context, thermal interference is not usually well known or addressed. Thermal interference is typically related to the presence or absence of a licensing system, procedures, management of existing data and an approach not based on the planning of installations, especially in dense urban areas. In such cases, interference problems can produce long term negative effects in systems operation.

A literature review highlighted three main aspects that are fundamental to preventing thermal interference problems: regulation, planning/management and technical aspects.

As a result of the review and data from the GEO4CIVHIC project, a 6-phase conceptual licensing flow chart was created. This diagram represents a guideline to identify weaknesses in the planning process and final realization of a BHE, giving suggestions on how to improve the specific (national, regional, or municipal) systems to prevent interference. The 6 steps are: (P1) the presence of a licensing system; (P2) the availability of an official GIS database; (P3) the technical optimisation and final design phase; (P4) the realization guarantee through certification of companies involved and the final checks from administration; (P5) the database update, to ensure consistent and reliable data and information for future installations; (P6) the monitoring that allows problems during operation to be identified and adjusted.

The 6-phase flow diagram ( Figure 2) was applied to the 16 real and virtual case studies from the GEO4CIVHIC project in different European countries. The assessment demonstrated how the different steps are addressed and allowed a score according to how many of the aspects of each phase were satisfied or present. The application of the conceptual licensing system model to the case studies showed that the optimisation and final design phase (P3) was the step which received the highest score. This result reflects the great effort performed by technicians and specialists to maximise the optimisation of a geothermal system during the dimensioning and planning steps (probably as a result of more national and international technical regulations). Unfortunately, even if the optimisation and final design are well implemented, the failures in the previous or following phases of the methodology can jeopardize the overall procedure, leading to potential future thermal interferences between different systems.

The analysis of the case studies led to the following general considerations:

- regulation requires the presence of centralized and simplified administrative processes, more structured authorisation, especially for large systems, and an integrated approach of different procedure levels (national/regional);
- an updated, public GIS platform, which stores and allows the visualization of collected data, including characteristics of specific installations and their thermal needs, would allow for better planning and management of existing and future installations;
- the use specific tools to simulate the long-term sustainability of the geothermal system before its realization in order to prevent interferences is important. These specific tools allow for the calculation and simulation of complex cases that can occur and that cannot be foreseen using simple “unwritten rules”.

The application of this 6-step model was useful to understand which aspects can cause critical thermal interference between geothermal systems in the analysed case studies. In this way, it is also possible to understand how they might be tackled in order to achieve optimal functioning systems in the long term.

The results of the case study analysis showed that one of the key shortcomings is the lack of information and data (location, length, number of boreholes drilled). An official database is usually missing and sometimes only the companies involved in the design and installation have detailed knowledge of these basic data. The final data in the database sometimes only shows the project data (and not the final implementation data). For this reason, the data might not show the right position and layout of BHE systems.

Through a reliable database, where information is digitised and geo-referenced in a public portal, the management of geothermal systems could be improved in the short term and in the future development of ground source heat pump systems. It is important that data are entered correctly during implementation because, unlike other renewable technologies such as photovoltaics, solar thermal, wind or hydro, which can be easily detected later e.g., using remote sensing or other techniques based on high-resolution image recognition, shallow geothermal systems are difficult to detect after installation due to their low visual impact. Policy should provide rules that favour such data collection and sharing to promote the long-term sustainable development of geothermal technology especially in dense urban spaces.

This 6-step conceptual model is a method that can be extended to all shallow geothermal systems for long-term planning. The methodology has to be applied considering the characteristic of each jurisdiction, the regulatory environment as well as national and local standards, the presence or absence of a database, stakeholder involvement and knowledge. The lack of a regulatory framework in some locations is one of the main barriers to successful implementation of geothermal energy systems. The application of the method allows a systematic approach to local regulatory frameworks to be applied and facilitates the identification of possible strengths or weaknesses in order to improve the local energy planning strategy in the context of ground source heat pump systems.

## Ethics and consent

Ethical approval and consent were not required

## Data Availability

This study involves a literature review and a case study which were used to present results in an existing report available from 14 and also from https://doi.org/10.3030/792355 (see section “Documents, reports”).
