# Supplementary material for: Application of a method for the sustainable planning and management of ground source heat pump systems in an urban environment, considering the effects of reciprocal thermal interference
Source: Open Res Eur. 2022 Nov 25;2:58. Originally published 2022 May 12. [Version 2] doi: 10.12688/openreseurope.14665.2 (PMC10445899; doi:10.12688/openreseurope.14665.2)
Supplement: Peer review history

# Peer review: Reviewer response for version 2

**DOI:** 10.21956/openreseurope.16468.r30421

**Published:** 2022-12-09

**Authors:** Alessandro Casasso

**Affiliations:** 1 Department of Environment, Land and Infrastructure Engineering (DIATI), Politecnico di Torino, Torino, Italy

The authors have provided the requested revisions and hence the article, from my point of view, is ready for being finalized and published.

Is the rationale for developing the new method (or application) clearly explained?

Partly

Is the description of the method technically sound?

Partly

Are the conclusions about the method and its performance adequately supported by the findings presented in the article?

Yes

If any results are presented, are all the source data underlying the results available to ensure full reproducibility?

Partly

Are sufficient details provided to allow replication of the method development and its use by others?

Partly

Reviewer Expertise:

Shallow geothermal energy, groundwater engineering

I confirm that I have read this submission and believe that I have an appropriate level of expertise to confirm that it is of an acceptable scientific standard.

# Peer review: Reviewer response for version 2

**DOI:** 10.21956/openreseurope.16468.r30423

**Published:** 2022-11-30

**Authors:** Jian Lin

**Affiliations:** 1 ICUBE, UMR 7357, CNRS, IUT Robert Schuman, University of Strasbourg, Strasbourg, France

Authors have considered the suggested modifications. The paper is now more concise.

Is the rationale for developing the new method (or application) clearly explained?

Partly

Is the description of the method technically sound?

Yes

Are the conclusions about the method and its performance adequately supported by the findings presented in the article?

Yes

If any results are presented, are all the source data underlying the results available to ensure full reproducibility?

Yes

Are sufficient details provided to allow replication of the method development and its use by others?

Partly

Reviewer Expertise:

Shallow geothermal systems

I confirm that I have read this submission and believe that I have an appropriate level of expertise to confirm that it is of an acceptable scientific standard.

# Peer review: Reviewer response for version 2

**DOI:** 10.21956/openreseurope.16468.r30422

**Published:** 2022-11-30

**Authors:** Cristina Sáez Blázquez

**Affiliations:** 1 Department of Cartographic and Land Engineering, Higher Polytechnic School of Ávila, Universidad de Salamanca, Ávila, Spain

Authors have correctly performed all the suggested changes.

Is the rationale for developing the new method (or application) clearly explained?

Yes

Is the description of the method technically sound?

Yes

Are the conclusions about the method and its performance adequately supported by the findings presented in the article?

Yes

If any results are presented, are all the source data underlying the results available to ensure full reproducibility?

Partly

Are sufficient details provided to allow replication of the method development and its use by others?

Partly

Reviewer Expertise:

Geothermal energy, shallow geothermal resources, renewable energy systems

I confirm that I have read this submission and believe that I have an appropriate level of expertise to confirm that it is of an acceptable scientific standard.

# Peer review: Reviewer response for version 1

**DOI:** 10.21956/openreseurope.15841.r29346

**Published:** 2022-07-05

**Authors:** Jian Lin

**Affiliations:** 1 ICUBE, UMR 7357, CNRS, IUT Robert Schuman, University of Strasbourg, Strasbourg, France

The manuscript presents the results of the GEO4CIVHIC project which aims to promote shallow geothermal systems. Based on the literature review, it proposes a conceptual model to prevent or limit long-term thermal interferences of borehole heat exchangers (BHEs). The proposed model has been applied to 16 sites in different European countries. Recommendations for the different steps of shallow geothermal projects have been given after the analysis of these case studies.

 In my view, the proposed conceptual model is convenient and practical to assess the planning of shallow geothermal projects. The 16 case studies and their source data provide a panorama of the shallow geothermal development situation in Europe. Yet, some parts of the manuscript structure needs to be improved and the authors should give more explications for the analysis procedure of case studies. Detailed suggestions are presented below:

1. The “Methods” section is dedicated to developing the methodology of the work but not the results. If the six phases of the conceptual model are considered as work results, they should not be presented here but in the following section.
2. Page 4, Figure 1: In the literature review step, as shown in Fig. 1, the number of consulted documents seems few for some types - among the nine total types, three types have only one document and three others have only two. Could the authors add some more consulted documents? If not, please justify it.
3. Page 4, Figure 2: Six steps of the proposed conceptual model have been shown here. It is recommended to separate the three steps before the installation of geothermal systems from three other steps after that (ex: by a line or with different colors).
4. Page 5: It is written in the text that “Specific software for simulation was already used to counteract the mutual influence in heat extraction”. Could the authors indicate the name(s) of the mentioned specific software(s)?
5. Page 6, “Application of the model to the real and virtual cases” section: Even if detailed information can be found in the project report (Ref. 14), the authors are recommended to explain how the percentage of each step was calculated from assessed scores of case studies from 0 to 3.
6. Page 7: Please indicate the percentage value of the “optimization and final design” phase (P3). And even if it could be evident, please explain its high value.

Is the rationale for developing the new method (or application) clearly explained?

Partly

Is the description of the method technically sound?

Yes

Are the conclusions about the method and its performance adequately supported by the findings presented in the article?

Yes

If any results are presented, are all the source data underlying the results available to ensure full reproducibility?

Yes

Are sufficient details provided to allow replication of the method development and its use by others?

Partly

Reviewer Expertise:

Shallow geothermal systems

I confirm that I have read this submission and believe that I have an appropriate level of expertise to confirm that it is of an acceptable scientific standard, however I have significant reservations, as outlined above.

# Response

**Published:** 2022-11-23

**Authors:** Marco Belliardi

**Affiliations:** SUPSI, Switzerland

Thank you for your positive evaluation and interest in our research. Thanks to your suggestions, we were able to improve our work in the following points:

- The title was changed from “A method for the sustainable…” to “Application of a method for the sustainable…” to highlight the fact that the results part is basically an application of a defined method to case studies, and it is intended to show which are the strongest and weakest points in the context of European case studies of the GEO4CIVHIC project;
- Figure 1 has been updated with new references, adding the timing information “before realization”, “realization”, “after realization”. This update has been applied also to Figure 2. New references have also been added and contextualised in the text;
- Some name of specific software (already used to counteract the mutual influence in heat extraction) for simulation has been mentioned;
- The complete calculation procedure that produced results in figure 5 has been described in detail, including the formulation and a table with “scores”. This is an important point and all reviewers agreed on the improvement of this part. In addition, a table was added to improve reproducibility and the calculation of scores. The authors really appreciate the comments and sincerely wish to have clarified and explained the elaboration;
- In the “conclusions” sections, an attempt was made to explain why phase 3 achieved a better result;
- In general, a complete revision of English was carried out including an improvement in the clarity of some sentences and concepts.

# Peer review: Reviewer response for version 1

**DOI:** 10.21956/openreseurope.15841.r29345

**Published:** 2022-06-17

**Authors:** Alessandro Casasso

**Affiliations:** 1 Department of Environment, Land and Infrastructure Engineering (DIATI), Politecnico di Torino, Torino, Italy

The paper analyses six key aspects of shallow geothermal plants design and authorisation, namely 1) licensing systems for the authorisation; 2) official databases of existing installations and of underground properties; 3) the design and optimization of the plant sizing; 4) the guarantee for a correct installation; 5) the update of the database (point no. 2); and 6) the monitoring phase.

 The paper is interesting and worth being published. However, there are some minor issues to be addressed:

- The abstract states the objectives of this paper (see "a) to analyze...c) to give technical...geothermal systems") that are not representative of its content, which focuses on how major phases of GSHP design and implementations are addressed.
- What do you mean by "official database"? The (geo-referenced) list of existing installations, mapping of underground properties, or of possible risks? Depending on your choice, the definition of this parameter for P2 (and P5) may differ considerably. Also, asking the plant designers to make all data used in the design phase publicly available is hardly feasible.
- The level of addressing of each phase (page 7 onwards), e.g. P1 58%: how is it calculated? Is there any % assigned to values from 0 to 3?
- Is there any "evaluation grid" for P1 to P6? For example, for P1 "the licensing system is available only for plants exceeding a certain power --> 2 points on 3", "not a real licensing system but more a notification system --> 1.5 points on 3". If not, a table like this would be of great help to understand the results of your analysis and for the replication of this method.
- What is a "virtual" case study? Please provide more details as this accounts for most of your case studies. Why did you choose "virtual" case studies instead of looking for more real ones?

Is the rationale for developing the new method (or application) clearly explained?

Partly

Is the description of the method technically sound?

Partly

Are the conclusions about the method and its performance adequately supported by the findings presented in the article?

Yes

If any results are presented, are all the source data underlying the results available to ensure full reproducibility?

Partly

Are sufficient details provided to allow replication of the method development and its use by others?

Partly

Reviewer Expertise:

Shallow geothermal energy, groundwater engineering

I confirm that I have read this submission and believe that I have an appropriate level of expertise to confirm that it is of an acceptable scientific standard, however I have significant reservations, as outlined above.

# Response

**Published:** 2022-11-23

**Authors:** Marco Belliardi

**Affiliations:** SUPSI, Switzerland

Thank you for your positive evaluation and interest in our research. Thanks to your suggestions, we were able to improve our work in the following points:

- Part of the abstract has been clarified especially concerning the revision of the objectives;
- The concept of official database was better explained;
- The complete calculation procedure that produced results in figure 5 has been described in detail, including the formulation and a table with “scores”. This is an important point and all reviewers agreed on the improvement of this part. In addition, a table was added to improve reproducibility and the calculation of scores. The authors really appreciate the comments and sincerely wish to have clarified and explained the elaboration;
- The difference between real and virtual case studies was clearer explained;
- In general, a complete revision of English was carried out including an improvement in the clarity of some sentences and concepts.

# Peer review: Reviewer response for version 1

**DOI:** 10.21956/openreseurope.15841.r29272

**Published:** 2022-06-16

**Authors:** Cristina Sáez Blázquez

**Affiliations:** 1 Department of Cartographic and Land Engineering, Higher Polytechnic School of Ávila, Universidad de Salamanca, Ávila, Spain

The research is interesting and valuable for the acceleration of efficient shallow geothermal systems in the EU context. In particular, it focuses on providing a methodology for planning an optimized GSHP installation, avoiding possible thermal interferences among systems.

 The information provided and the scope of the paper are of vital importance for the geothermal field and the correct development of shallow resources. The conceptual model for the system planning is accordingly described, but I miss some specific results of the evaluations performed in the areas under study. There is just a figure (Figure 5) with undetailed data about the application of the model in the case studies, but there is no clear information about it. In my opinion, there are not sufficient details for the reader to understand the possible replication of the methodology, at least with the information provided in the paper.

 The results of the implementation of the method would be of real interest to the general geothermal community and they could be commented on in a more detailed way in the manuscript. Despite the link provided by the authors including the information on the project, they should have shown some of them in the paper. In this way, the results presented out of the paper (through the provided link) ensure the full reproducibility of the work, including all the deliverables and reports, patents, and additional publications obtained from the project.

 Finally, the conclusion section is well described and presented.

Is the rationale for developing the new method (or application) clearly explained?

Yes

Is the description of the method technically sound?

Yes

Are the conclusions about the method and its performance adequately supported by the findings presented in the article?

Yes

If any results are presented, are all the source data underlying the results available to ensure full reproducibility?

Partly

Are sufficient details provided to allow replication of the method development and its use by others?

Partly

Reviewer Expertise:

Geothermal energy, shallow geothermal resources, renewable energy systems

I confirm that I have read this submission and believe that I have an appropriate level of expertise to confirm that it is of an acceptable scientific standard, however I have significant reservations, as outlined above.

# Response

**Published:** 2022-11-23

**Authors:** Marco Belliardi

**Affiliations:** SUPSI, Switzerland

Thank you for your positive evaluation and interest in our research. Thanks to your suggestions, we were able to improve our work in the following points:

- The complete calculation procedure that produced results in figure 5 has been described in detail, including the formulation and a table with “scores”. This is an important point and all reviewers agreed on the improvement of this part. The authors really appreciate the comments and sincerely wish to have clarified and explained the elaboration
- In order to ensure the full reproducibility of the work, in addition to the wording described above, a table was added with the scores awarded (based on a clearer description of the calculation as you suggested)
- In general, a complete revision of English was carried out including an improvement in the clarity of some sentences and concepts.
